# Nuclease-resistant immunostimulatory phosphodiester CpG oligodeoxynucleotides as human Toll-like receptor 9 agonists

**DOI:** 10.1186/1472-6750-11-88

**Published:** 2011-09-26

**Authors:** Wenjun Meng, Tomohiko Yamazaki, Yuuki Nishida, Nobutaka Hanagata

**Affiliations:** 1 Graduate School of Life Science, Hokkaido University, N10W8, Kita-ku, Sapporo 060-0810, Japan; 2 International Center for Materials Nanoarchitectonics (MANA), National Institute for Materials Science (NIMS), 1-2-1 Sengen, Tsukuba, Ibaraki 305-0047, Japan; 3 Interdisciplinary Laboratory for Nanoscale Science and Technology, NIMS, 1-2-1 Sengen, Tsukuba, Ibaraki 305-0047, Japan

## Abstract

**Background:**

Unmethylated cytosine-guanine (CpG) motif-containing oligodeoxynucleotides (ODNs) have been well characterized as agonists of Toll-like receptor 9 (TLR9). ODNs with a phosphorothioate (PTO) backbone have been studied as TLR9 agonists since natural ODNs with a phosphodiester (PD) backbone are easily degraded by a serum nuclease, which makes them problematic for therapeutic applications. However, ODNs with a PTO backbone have been shown to have undesirable side effects. Thus, our goal was to develop nuclease-resistant, PD ODNs that are effective as human TLR9 (hTLR9) agonists.

**Results:**

The sequence of ODN2006, a CpG ODN that acts as an hTLR9 agonist, was used as the basic CpG ODN material. The 3'-end modification of ODN2006 with a PD backbone (PD-ODN2006) improved its potential as an hTLR9 agonist because of increased resistance to nucleolytic degradation. Moreover, 3'-end modification with oligonucleotides showed higher induction than modification with biotin, FITC, and amino groups. Further, enhancement of hTLR9 activity was found to be dependent on the number of CpG core motifs (GTCGTT) in the PD ODN containing the 3'-end oligonucleotides. In particular, ODN sequences consisting of two to three linked ODN2006 sequences with a PD backbone (e.g., PD-ODN2006-2006 and PD-ODN2006-2006-2006) acted as effective agonists of hTLR9 even at lower concentrations.

**Conclusions:**

This study showed that PD-ODN2006-2006 and PD-ODN-2006-2006-2006 can be used as potentially safe agonists for hTLR9 activation instead of CpG ODNs with a PTO backbone. We propose these CpG ODNs consisting of only a PD backbone as a novel class of CpG ODN.

## Background

Unmethylated cytosine-phosphate-guanine (CpG) motifs are considered to be pathogen-associated molecular patterns because of their abundance in microbial genomes but scarcity in vertebrate ones [1]. Short, single-stranded, synthetic oligodeoxynucleotides with CpG motifs (CpG ODNs) can induce the Th1-type immune response through interaction with Toll-like receptor 9 (TLR9), a member of the Toll-like family of pattern-recognition receptors [2-4]. Because Th1-type cytokines such as IFN-γ and IL-12 can inhibit the Th2-type response [5,6], CpG ODNs are thought to have potential for various immune therapies such as for cancer, asthma, pollinosis, and infectious diseases [7-9].

To date, at least four different types of immune-stimulatory CpG ODNs with distinct structural and biological characteristics have been reported [10]. The class A CpG ODNs consist of phosphodiester (PD), CpG-containing, palindromic motifs with phosphorothioate (PTO), poly-G motifs at both the 5' and 3' ends. CpG ODNs in this class can induce high levels of interferon-α (IFN-α) production in plasmacytoid dendritic cells (pDCs), but are weak stimulators of TLR9-dependent NF-κB signaling [11]. On the other hand, the class B CpG ODNs consist entirely of PTO backbones and do not form palindromic structures. These ODNs can strongly activate B cells through NF-κB signaling but show no effect on IFN-α production [12,13]. The class C CpG ODNs show an intermediate immune property between classes A and B, since these CpG ODNs have CpG-containing palindromic motifs consisting of only a PTO backbone [14]. Recently, a novel type of CpG ODN called the P class, which has two palindromic motifs with a PTO backbone, was reported to show higher potential to produce IFN-α and activate NF-κB [15].

Natural PD-ODNs are susceptible to nuclease degradation, which renders them inactive for TLR9 activation in the free form. Therefore, all CpG ODNs classified above partially or completely contain a PTO backbone to increase resistance to nuclease degradation in the serum [16-18]. However, the PTO backbone has been reported to cause unwanted side effects such as unspecific binding to various proteins [19] and renal damage [20]. Therefore, because of safety considerations, the development of novel natural CpG ODNs consisting of an entirely PD backbone is desirable. Furthermore, these natural CpG ODNs should be able to strongly activate TLR9-dependent NF-κB signaling. To develop such a CpG ODN, we examined the effects of the sugar backbone, the sequence of the CpG motif, and modification of both terminals in CpG ODNs on NF-κB activation. We found that a series of linked PD sequences of ODN2006 (a class B agonist of hTLR9) has the potential to resist nuclease degradation and to activate NF-κB through TLR9 at even lower concentrations than are known for PTO ODNs.

## Results

### Natural CpG ODNs consisting of a PD backbone have no potential to activate TLR9 because of degradation

**Figure 1:**
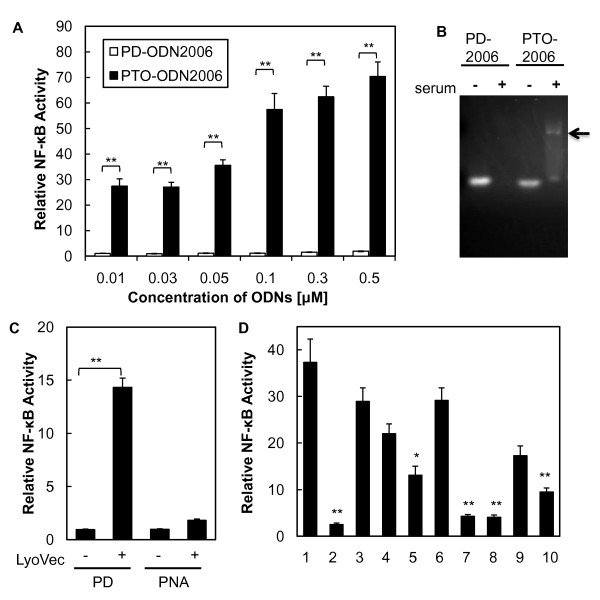
**Effect of ODN backbones and CpG motif on NF-κB activation**. (A) 293XL-hTLR9 cells transfected with hTLR9-signaling reporter plasmid were stimulated by PD-ODN2006 and PTO-ODN2006 with various concentrations. n = 3, mean (sd). Statistical significances are denoted as follows: *P < 0.05, **P < 0.01. (B) Stability of PD-ODN2006 and PTO-ODN2006 in aqueous solution with/without serum. The arrow indicates PTO-ODN-protein complex. (C) PD-ODN2006 and PNA-ODN2006 (0.2 μM) were applied to cells with/without LyoVec. n = 3, mean (sd). Statistical significances are denoted as follows: *P < 0.05, **P < 0.01. (D) 0.1 μM of PTO-ODN2006 mutants (1: PTO-ODN2006, 2: PTO-ODN2006-GC, 3: PTO-ODN2006-1GC, 4: PTO-ODN2006-2GC, 5: PTO-ODN2006-3GC, 6: PTO-ODN2006-4GC, 7: PTO-ODN2006-2,3,4GC, 8: PTO-ODN2006-2,3GC, 9: PTO-ODN2006-2,4GC, 10: PTO-ODN2006-3,4GC) were applied to cells. n = 3, mean (sd). All *P *values are shown relative to the PTO-ODN2006, *P < 0.05, **P < 0.01.

To develop natural CpG ODNs with a PD backbone that are capable of strong human TLR9 activation, the ODN2006 sequence, a well-known class B agonist of hTLR9 [13], was used as a basic sequence for designing CpG ODNs. TLR9 activation was assessed by relative NF-κB activation in human TLR9-expressing cells (293XL-hTLR9), since TLR9 activation by CpG ODNs leads to activation of NF-κB [21]. We synthesized an ODN2006 sequence consisting of either a PD or PTO backbone (PD-ODN2006 and PTO-ODN2006, respectively) (Table 1) and examined the effect of the backbone composition on NF-κB activity. PD-ODN2006 showed little potential for activating TLR9, as demonstrated by the increased NF-κB activity, whereas PTO-ODN2006 significantly activated NF-κB in a dose-dependent manner (Figure 1A). Identical to previous reports on the susceptibility of natural PD ODNs to nuclease degradation [16-18], PD-ODN2006 likely showed low potential for activating TLR9 because of degradation in the serum (Figure 1B). In contrast, PTO-ODN2006 still remained as clear band after serum treatment, but a high-molecular-weight band was also observed (Figure 1B). This band could be attributable to proteins binding to the PTO backbone rather than being specifically associated with the PTO-ODN2006 sequences, since Brown et al. [19] also reported binding of proteins to ODNs with PTO backbones. Our result suggests that PTO-ODN2006 resists nuclease degradation, but that an unfavorable reaction may also be occurring in the serum. Therefore, novel GpG ODNs that can stimulate TLR9 activity need to resist nucleolytic degradation as well as not react with serum proteins.

**Table 1:** Oligodeoxynucleotide sequences examined in this study

ODNs	Sequences (5'→3')
PD-ODN2006	**TCGTCGTTTTGTCGTTTTGTCGTT**
PTO-ODN2006	tcgtcgttttgtcgttttgtcgtt
PTO-ODN2006-GC	t**gc**t**gc**ttttgt**gc**ttttgt**gc**tt
PTO-ODN2006-1GC	t**gc**tcgttttgtcgttttgtcgtt
PTO-ODN2006-2GC	tcgt**gc**ttttgtcgttttgtcgtt
PTO-ODN2006-3GC	tcgtcgttttgt**gc**ttttgtcgtt
PTO-ODN2006-4GC	tcgtcgttttgtcgttttgt**gc**tt
PTO-ODN2006-2,3,4GC	tcgt**gc**ttttgt**gc**ttttgt**gc**tt
PTO-ODN2006-2,3GC	tcgt**gc**ttttgt**gc**ttttgtcgtt
PTO-ODN2006-2,4GC	tcgt**gc**ttttgtcgttttgt**gc**tt
PTO-ODN2006-3,4GC	tcgtcgttttgt**gc**ttttgt**gc**tt
PD-ODN2006-3'-A	**TCGTCGTTTTGTCGTTTTGTCGTT**-CCTTCAGTGGGACC
PD-ODN2006-5'-A	CCTTCAGTGGGACC-**TCGTCGTTTTGTCGTTTTGTCGTT**
PD-ODN2006-3'-B	**TCGTCGTTTTGTCGTTTTGTCGTT-**GGTCCCACTGAAGG
PD-ODN2006-5'-B	GGTCCCACTGAAGG-**TCGTCGTTTTGTCGTTTTGTCGTT**
PD-ODN2006-3'-A-2006	**TCGTCGTTTTGTCGTTTTGTCGTT**-CCTTCAGTGGGACC-**TCGTCGTTTTGTCGTTTTGTCGTT**
PD-ODN2006-3'-B-2006	**TCGTCGTTTTGTCGTTTTGTCGTT**-GGTCCCACTGAAGG-**TCGTCGTTTTGTCGTTTTGTCGTT**
PD-ODN2006-2006	**TCGTCGTTTTGTCGTTTTGTCGTT-TCGTCGTTTTGTCGTTTTGTCGTT**
PD-ODN2006-TCGTCGTT	**TCGTCGTTTTGTCGTTTTGTCGTT**-TCGTCGTT
PD-ODN2006-TCGTCGTTTTGTCGTT	**TCGTCGTTTTGTCGTTTTGTCGTT-**TCGTCGTTTTGTCGTT
PD-ODN2006-2006-2006	**TCGTCGTTTTGTCGTTTTGTCGTT-TCGTCGTTTTGTCGTTTTGTCGTT-TCGTCGTTTTGTCGTTTTGTCGTT**
PD-ODN2006-G5	**TCGTCGTTTTGTCGTTTTGTCGTT**-GGGGG

ODNs: oligodeoxynucleotides; PD: phosphodiester; PTO: phosphorothioate. Capital and lowercase letters in sequences indicate PD and PTO backbones, respectively. Bold sequences indicate the PD-ODN2006 sequence.

### Peptide nucleic acid inhibits TLR9 activation

Peptide nucleic acids (PNAs), which have backbones composed of repeated N-(2-aminoethyl)-glycine units linked by peptide bonds, have been developed to solve the problem of biological instability in short-stranded DNA or synthetic ODNs [22]. Thus, we examined the potential of ODN2006 consisting of a peptide backbone (PNA-ODN2006) for TLR9 activation. However, no enhancement of TLR9 activity was observed (Figure 1C). Interestingly, PD-ODN2006 showed higher potential when LyoVec, a cationic lipid, was used as a carrier to prevent nucleolytic degradation, but the carrier had no effect for PNA-ODN2006. This indicates that the lack of TLR9 activation by PNA-ODN2006 is attributable to PNA and that the sugar backbone of CpG ODN is essential for TLR9 activation.

### CpG ODNs activate TLR9 in a sequence-dependent manner

Next, we tried to design novel CpG ODN sequences with a higher potential for activating TLR9 than ODN2006. To determine if mutation or deletion of CG units could improve CpG ODN design, we prepared different mutants of PTO-ODN2006 (Table 1) by replacing CG unit(s) with GC unit(s) and then examined their potential. NF-κB activity was dramatically decreased by PTO-ODN2006-GC, an ODN in which all four CG units had been replaced with GC ones (Figure 1D), indicating the importance of the CG units for TLR9 activation. Interestingly, the third CG unit in PTO-ODN2006 appeared to be the most important one, since lower activity was observed for PTO-ODN2006-3GC than for the three other ODNs with a single GC substitution (Figure 1D). Similarly, lower activity was also observed for PTO-ODN2006-2,3,4GC, PTO-ODN2006-2,3GC, and PTO-ODN2006-3,4GC, than for PTO-ODN2006-2,4GC. This suggests that the position and number of CG units in ODN2006 are critical for TLR9 activation, and that neither mutation nor deletion of the CG units can enhance activity.

### Modification at the 3'-end of PD-ODN2006 improves TLR9 activation

**Figure 2:**
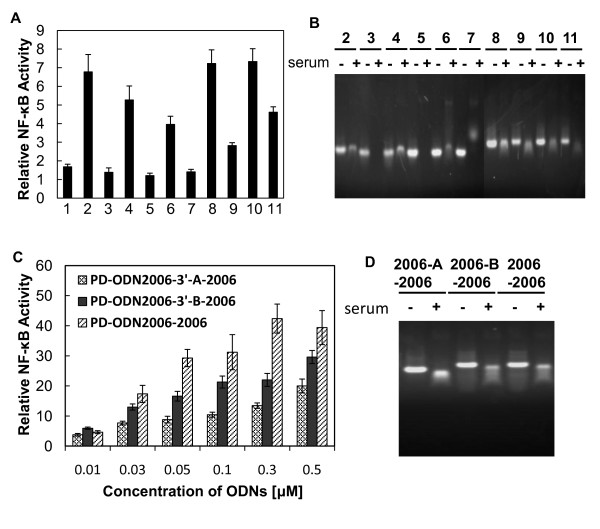
**Effect of terminal modification and linker sequences in PD-ODN2006 on NF-κB activation**. (A) 293XL-hTLR9 cells transfected with hTLR9-signaling reporter plasmid were stimulated by modified PD-ODN2006s (1: PD-ODN2006, 2: PD-ODN-3'-Biotin, 3: PD-ODN-5'-Biotin, 4: PD-ODN-3'-FITC, 5: PD-ODN-5'-FITC, 6: PD-ODN-3'-NH_2_, 7: PD-ODN-5'-NH_2_, 8: PD-ODN-3'-A, 9: PD-ODN-5'-A, 10: PD-ODN-3'-B, 11: PD-ODN-5'-B) at a concentration of 0.5 μM. n = 3, mean (sd). All *P *values are shown relative to the PD-ODN2006, *P < 0.05, **P < 0.01. (B) Stability of modified PD-ODN2006s in aqueous solution with/without serum. (C) 293XL-hTLR9 cells transfected with hTLR9-signaling reporter plasmid were stimulated by series connection of PD-ODN2006 with/without linker sequences. n = 3, mean (sd). (D) Stability of these PD-ODNs in aqueous solution with/without serum.

Exonucleases cleave nucleotides one at a time from the terminal end of a polynucleotide chain. Thus, we modified the PD-ODN2006 5' and 3' terminal ends using biotin, FITC, and amino groups (-NH_2_) to examine whether these changes would improve the potential for TLR9 activation. As shown in Figure 2A, modifications at the 3'-end of PD-ODN2006 enhanced NF-κB activity relative to no modification of PD-ODN2006. However, no significant change was observed by modifying the 5'-end. To clarify why the 3'-modifications improved the activity, we incubated the 3'-modified PD-ODN2006s in serum and found that they showed improved resistance to degradation (Figure 2B).

We further modified the PD-ODN2006s by adding two 14-mer oligonucleotides (called A and B) consisting of a PD backbone (PD-ODN2006-5'/3'-A and PD-ODN2006-5'/3'-B, respectively) (Table 1). These ODNs showed enhanced NF-κB activity relative to the unmodified PD-ODN2006 (Figures 2A and 2B). Interestingly, modification at the 5'-end also improved activity (Figure 2A). This seemed to be due to the presence of CG units remaining after degradation, since molecules were detected even after incubation in serum (Figure 2B).

### PD-ODN2006 series acts as a novel type of TLR9 agonist

Since the addition of the 14-mer oligonucleotides to the 3'-end of PD-ODN2006 significantly improved stability and led to increased NF-κB activity, we examined the effect of adding other oligonucleotide sequences to the 3'-end of PD-ODN2006. Three types of modified PD-ODN2006 sequences were designed. PD-ODN2006-3'-A-2006 and PD-ODN2006-3'-B-2006 were constructed from an ODN2006 sequence extension at the 3'-end of PD-ODN2006-3'-A and PD-ODN2006-3'-B, respectively (Table 1). For these ODNs, oligonucleotides A and B were linkers between the two PD-ODN2006s. As the third modified sequence, PD-ODN2006-2006 comprised two directly linked PD-ODN2006s. All three structures increased NF-κB activity in a dose-dependent manner (Figure 2C). Although PD-ODN2006-3'-A-2006 and PD-ODN2006-3'-B-2006 enhanced activity to a further extent compared to PD-ODN2006-3'-A and PD-ODN2006-3'-B at the same concentration (0.5 μM) (see Figure 2A), PD-ODN2006-2006 was more effective than PD-ODN2006-3'-A-2006 and PD-ODN2006-3'-B-2006 at every concentration. All three 3'-modified PD-ODN2006s with oligonucleotides resisted nucleolytic degradation as well as the other 3'-end modified PD-ODN2006 (Figure 2D).

**Figure 3:**
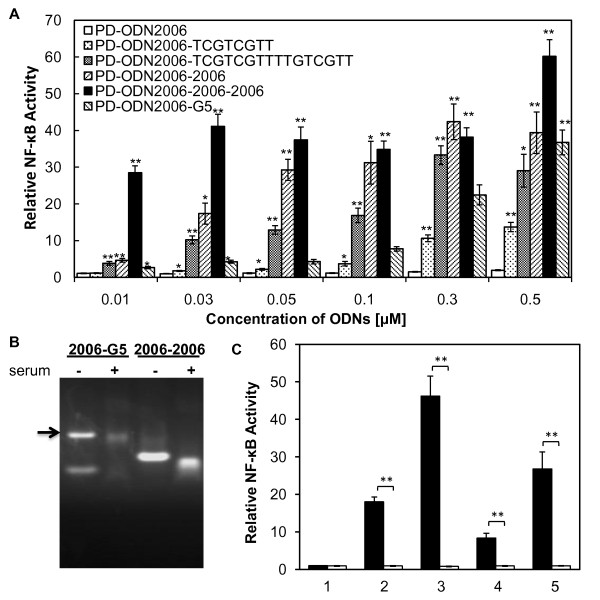
**Effect of novel CpG-ODNs on NF-κB activation in 293XL-hTLR9 cells and 293XL-null cells**. (A) 293XL-hTLR9 cells transfected with hTLR9-signaling reporter plasmid were stimulated by PD-ODN with an elevated number of CpG motifs. n = 3, mean (sd). All *P *values are shown relative to the PD-ODN2006 with the same concentrations, *P < 0.05, **P < 0.01. (B) Stability of PD-ODNs in aqueous solution with/without serum. The arrow indicates supramolecular structure of PD-ODN2006-G5. (C) NF-κB activation by PD-ODNs (1: PD-ODN2006, 2: PD-ODN2006-2006, 3: PD-ODN2006-2006-2006, 4: PD-ODN2006-G5, 5: PTO-ODN2006) in hTLR9 deficient cells (293XL-null) and hTLR9 expressing cells (293XL-hTLR9 cells). Statistically significant results are denoted as follows: *P < 0.05, **P < 0.01.

It has been reported that the GTCGTT sequence is a core CpG motif for hTLR9 activation [23]. Thus, the effect of the 3'-end extension of PD-ODN2006 by this core motif was examined. PD-ODN2006-3'-TCGTCGTT and PD-ODN2006-3'-TCGTCGTTTTGTCGTT contain one and two core motif(s) at the 3' end, respectively (Table 1). As the PD-ODN2006 sequence contains three core motifs, these CpG ODNs represented a total of four and five core motifs. PD-ODN2006-2006 and PD-ODN2006-2006-2006 also have six and nine core motifs, respectively (Table 1). As shown in Figure 3A, the potential for TLR9 activation depended on the number of core motifs. In particular, PD-ODN2006-2006-2006 enhanced the activity even at a concentration of 0.01 μM. Moreover, PD-ODN2006-2006 showed much higher potential than PD-ODN2006-3'-TCGTCGTTTTGTCGTT at concentrations below 0.1 μM.

Linked series of PD-ODN2006 (e.g., PD-ODN2006-2006-2006) also showed higher potential than that reported for PD-ODN2006-G5, the backbone of which consists of PD entirely [24,25]. These differences were especially significant at the lower concentrations (Figure 3A). A higher molecular band was observed for PD-ODN2006-G5, which may be attributable to a supramolecular structure formed by the poly-G tails [26]. However, none were observed for the ODNs consisting of PD-ODN2006 in a series (Figure 3B). To examine whether NF-κB activation by PD-ODNs is mediated by TLR9, we used 293XL-null cells. These TLR9-deficient cells did not show any significant increase in NF-κB activity when stimulated either by PTO-ODN2006 or our novel PD-ODNs (Figure 3C).

**Figure 4:**
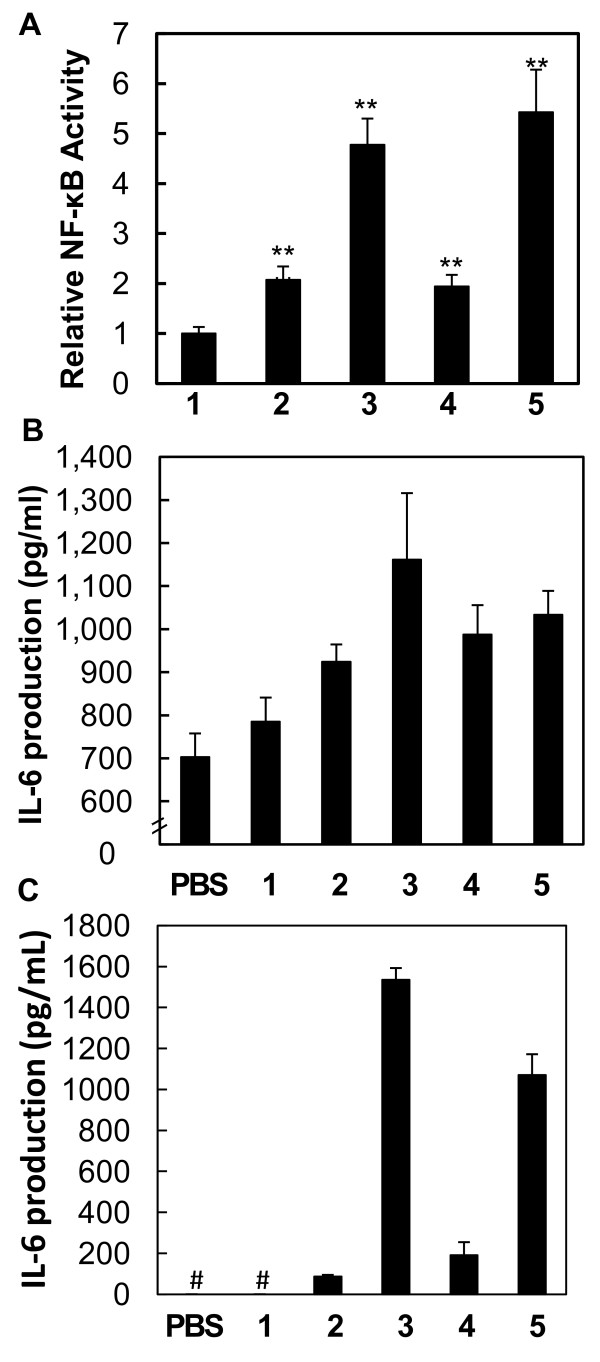
**TLR9 activation by B lymphocytes, PBMC, and CAL-1 cells**. (A) Activation of NF-κB in human B lymphocyte cell lines, Ramos-Blue cells, stably transfected with NF-kB/AP-1-inducible SEAP reporter gene. n = 3, mean (sd). All P values are shown relative to the PD-ODN2006: *P < 0.05, **P < 0.01. (B-C) IL-6 production induced by CpG-ODNs (1: PD-ODN2006, 2: PD-ODN2006-2006, 3: PD-ODN2006-2006-2006, 4: PD-ODN2006-G5, 5: PTO-ODN2006). n = 3, mean (sd). From human PBMCs (B) and human plasmacytoid dendritic cells CAL-1(C). The symbol # indicates that no cytokine secretion was detected.

We also assessed the potential of PD-ODNs using B lymphocyte Ramos-Blue cells. PD-ODN2006-2006 had similar potential to PD-ODN2006-G5, but less than PTO-ODN2006. However, the potential of PD-ODN2006-2006-2006 was much higher than that of PD-ODN2006-G5 and almost the same as PTO-ODN2006 (Figure 4A). In addition, PD-ODN2006-2006-2006 had higher potential to induce IL-6 secretion in peripheral blood mononuclear cells (PBMCs) than PD-ODN2006-G5 and PTO-ODN2006, although no significant change was observed (Figure 4B). We also examined the IL-6 secretion in plasmacytoid dendritic cells (CAL-1). For PD-ODN2006-2006-2006, the secretion of IL-6 was higher than PD-ODN2006-G5 and PTO-ODN 2006, which was consistent with what was observed in the PBMCs. However, the secretion of IL-6 by the stimulation of PBS and PD-ODN2006 was not detected in this cell line (Figure 4C).

## Discussion

Synthetic oligonucleotides in recent use are usually protected from degradation by modification of the backbone, whereas natural CpG ODNs are readily degraded by ubiquitous nucleases both *in vivo *and *in vitro *[16-18]. The CpG ODNs developed in this study consist of only a natural PD backbone, which may eliminate the side effects introduced by the PTO backbone modification. Moreover, by connecting two or three simple ODNs, we obtained novel CpG ODNs with better nuclease tolerance and higher immunostimulatory potential in hTLR9-dependent NF-κB activation.

We used the sequence of a typical class B CpG ODN (ODN2006) to develop novel agonists for human TLR9. In our *in vitro *model system, it was shown that natural PD-ODN2006 likely had very little potential for activating NF-κB due to degradation by serum nucleases. TLR9 activation requires the internalization of CpG ODNs into the late endosome/lysosome of TLR9-expressing cells for recognition. Therefore, protection of the ODNs from degradation is an initial step in maintaining their activity. Hence, PTO backbones are commonly used to protect the open ends of linear oligonucleotides. Although PTO-ODN2006 demonstrated much higher potential for TLR9 activation than PD-OD2006, it was not a perfect candidate as an immunotherapeutic agent. The PTO backbone modification led to nonspecific interactions between PTO-ODN2006 and serum proteins, as shown with the high-molecular-weight band for this ODN, whereas no bands were obtained for PD-ODN2006. This result also suggests that the side effects of PTO-ODNs may be a product of the backbone modification and may not be related to the increased half-life.

PNA is a DNA analog in which the natural phosphodiester backbone of DNA is replaced by a 2-aminoethyl-glycine linkage. PNA modification is widely used to increase the biological stability of short-stranded DNA when developing antisense technology [22]. We examined the effect of the PNA-ODN2006 sequence, but no significant increase in activity was observed. Haas et al. suggested that the DNA sugar backbone (2' deoxyribose) determines TLR9 activation because they found that the corresponding RNA ribo-oligonucleotides could not activate TLR9 [27]. In our study, the inhibitory effect of PNA-ODN also supports the suggestion that the 2' deoxyribose in the DNA plays a crucial role in TLR9 activation.

We concluded from the decreased activity of the mutated PTO-ODN2006 that all the CG units in ODN2006 were essential for activation. Moreover, the third CG seemed to be the most important unit for TLR9 activation. Hence, protection of the CG units from degradation in natural PD-ODN2006 is crucial for maintaining its immunostimulatory activity. Modification of ODN2006 with either organic molecules at the 3'-end or oligonucleotides at the 3'- or 5'-ends enhanced the stimulatory activity. This enhancement can be explained by the increased resistance of the ODNs to nuclease degradation. Although it has been reported that free 5' ends in CpG ODNs with a PTO backbone are required for the activation of TLR9 [28,29], loss of activity in 5'-modified PD-ODN2006 in this study was attributed to degradation. When we modified PD-ODN2006 with 14-mer oligonucleotides at the 5'-end, there was still some potential to activate NF-κB, with some bands still present after serum degradation.

To further increase the immunostimulatory activity of the 3'-modified PD-ODN2006, we next prepared a series of linked PD-ODN2006 sequences (PD-ODN2006-3'-A-2006 and ODN2006-3'-B-2006), which increased the number of CG motifs in each oligonucleotide. Surprisingly, PD-ODN2006-3'-B-2006 led to higher activity than PD-ODN2006-3'-A-2006 at every concentration, which indicated that the linker sequence can affect the potential of CpG ODNs for NF-κB activation. Finally, we removed the linker, generating PD-ODN2006s such as PD-ODN2006-2006 and PD-ODN2006-2006-2006. The enhanced potential for NF-κB activation obtained by CpG-ODN2006-2006 compared with ODN2006-A/B-2006 demonstrated that the linker sequences used in this study inhibited the activation of TLR9. Moreover, PD-ODN2006-2006-2006 showed much higher activity than PD-ODN2006-2006, indicating that the number of CG motifs played a key role in the activity. To verify that the ODN2006-linked sequences activate NF-κB activation through the TLR9 pathway, we applied PD-ODN2006-2006 into a 293XL-null cell line that does not express hTLR9. Consequently, we observed no NF-κB activation in this cell line. Therefore, PD-ODN2006-2006 is thought to indeed activate NF-κB through the TLR9 pathway. Hence, these series of PD-ODN2006 sequences might be used as novel and effective TLR9 agonists.

To our knowledge, there are a few reports on nuclease-resistant immunostimulatory CpG ODNs with entirely PD backbones. Lee et al. [25] reported that the conjugation of poly-guanosine (poly-G) sequences at the 3'-end of PD-CpG ODNs significantly enhanced TNF-α and IL-12 production from mouse splenic dendritic cells. Bartz et al. [26] modified PD-ODN2006 with poly-G sequences and increased the cellular uptake and stimulatory activity of PD-ODN2006 in human leukocytes. They suggested that the increase in the activity resulted from the enhanced cellular uptake, which was attributed to the formation of ODN tetrads, G-Quadra structure. We also observed a supramolecular structure for PD-ODN2006-G5, as indicated by the presence of a high-molecular-weight band from agarose electrophoresis. However, no high-molecular-weight aggregate was formed for PD-ODN2006-2006. Atomic force microscopy also revealed that class A CpG ODNs with poly-G sequences form a particle structure, while class B PTO-ODN2006 forms a linear structure [30]. Clear bands of PD-ODN2006-2006 and PD-ODN-2006-2006-2006 remained after serum degradation, indicating that the series of natural PD-ODN2006s preserved the potential for TLR9 activation due to enhanced stability. We also speculate that the increase in the number of CG motifs in the oligonucleotides played an important role in the enhanced TLR9 activation potential. Our novel PD-ODNs demonstrated higher NF-κB activity in not only the 293XL-hTLR9 model cell lines but also B lymphocyte cell lines (Ramos-Blue) when stimulated with PD-ODN2006-2006 and PD-ODN2006-2006-2006.

In this study, we examined the production level of IL-6 because our CpG ODNs are considered to have the same linear structure, which is proved to activate only B cells but not dendritic cells that produce IL-12 and type I interferon, as class B CpG ODNs [12,13]. Our PD-ODNs also showed a higher potential for the production of IL-6. Therefore, our CpG-ODNs are suitable for immunotherapeutic use.

## Conclusion

The purpose of this study was to develop safe and effective CpG ODNs consisting of a PD backbone for hTLR9 activation. Our data indicate that 3'-modified CpG ODNs consisting of a PD backbone are effective at resisting nucleolytic degradation, and that the number of CpG core motifs strongly influence their potential as hTLR9 agonists. In particular, CpG ODNs with linked PD-ODN2006 sequences (e.g., PD-ODN2006-2006 and PD-ODN-2006-2006-2006) can act as effective and potentially safe agonists of hTLR9 at low concentrations.

## Methods

### Cell cultures

293XL-hTLR9 cells stably expressing hTLR9 and 293XL-null cells were purchased from Invivogen (CA, US). Cells were grown in high-glucose, Dulbecco-modified Eagle's medium (DMEM) supplemented with 10% fetal bovine serum (FBS), 50 units/mL penicillin, 50 mg/mL streptomycin, and 10 μg/mL blasticidin at 37°C in humidified air containing 5% CO_2_. Cells were seeded in 48-well culture plates for transfection and cell stimulation. A B lymphocyte cell line that stably expresses an NF-kB/AP-1-inducible secreted embryonic alkaline phosphate (SEAP) reporter gene, Ramos-Blue cell line (Invivogen), was cultured in Iscove's Modified Dulbecco's Medium (IMDM) supplemented with 10% (FBS), 2 mM L-glutamine, 50 units/mL penicillin, and 50 mg/mL streptomycin. Peripheral blood mononuclear cells (PBMCs) were purchased from Cellular Technology Limited (OH, US) as frozen cells. The procedures for the thawing of PBMCs were adapted from the manufacturer's protocol. The plasmacytoid dendritic cell line, CAL-1 was maintained in RPMI 1640 medium supplemented with 10% FBS as indicated in the report [31].

### ODNs

ODNs consisting of phosphodiester (PD) and phosphorothioate (PTO) backbones, and ODNs modified with biotin, FITC, or amino groups (-NH2) were purchased from Fasmac Inc. (Kanagawa, Japan). The ODNs were diluted in sterilized water and stored at -20°C. Peptide nucleic acid was purchased from Panagene (Daejeon, Korea).

### NF-κB luciferase assay

To monitor transient NF-κB activation, 293XL-hTLR9 cells and 293XL-null cells were seeded at 1 × 10^5 ^cells per well and transiently transfected with pNiFty-luc (a TLR9-signaling reporter plasmid, Invivogen) and pGL4.74 (a Renilla luciferase gene containing plasmid, Promega, WI, US), using LyoVec (Invivogen). After 24 h, cells were stimulated with oligonucleotides. These cells were lysed using a passive lysis buffer after 24 h, and lysates were assayed for luciferase activity using a luminometer (TD-20/20, Promega) according to the manufacturer's instructions. The data were represented as relative NF-κB activity compared with that of non-stimulated control cells.

B lymphocyte Ramos-Blue cells were seeded at a density of 2 × 10^6 ^cells/mL, and the cells were immediately stimulated with 0.5 μM ODNs. Cell supernatants were collected, and NF-κB activation was examined by the reporter gene SEAP expression levels using a spectrophotometer at 620 nm.

### Stability of oligodeoxynucleotides in serum

Twenty microliters of ODNs at a final concentration of 10 μM was incubated in an aqueous solution containing 20% FBS at 37°C for 16 h. All samples were subsequently treated with 2 μL of 250 mM EDTA for 2 min at 80°C to quench the digestion reaction. The integrity of the ODNs was then analyzed by gel electrophoresis using a 4% agarose gel.

### Measurement of IL-6 release from PBMC and CAL-1

After thawing, PBMCs were seeded in serum free medium, CTL-test medium (Cellular Technology Limited), at a density of 1.0 × 10^6 ^cells/mL. Cells were immediately stimulated with 0.5 μM ODNs. After 24 h of incubation at 37°C, cell supernatants were collected and stored at -20°C until further analysis. CAL-1 was seeded at a density of 5.0 × 10^6 ^cells/mL and stimulated with 1.5 μM ODNs for 48 hours. Supernatants were collected and stored at -20°C until further analysis. The level of human IL-6 in the media was determined by enzyme-linked immunosorbent assay (ELISA) using the Ready-SET-Go! Set (eBiosciences, San Diego, CA). The procedures for the ELISA for IL-6 were adapted from the manufacturer's protocol.

### Statistical Analysis

Stoic analysis was performed using Student's t-test. A *p*-value less than 0.05 was considered to be statistically significant.

## Authors' contributions

WM carried out most of the experiments including the NF-κB luciferase assay and DNA degradation experiments, performed the statistical analysis, and drafted the manuscript. YT participated in the design of the study and the statistical analysis, contributed to most of the experiments and helped draft the manuscript. NY participated in the preparation of plasmids and cell maintenance. HN initiated and supervised the study and revised and finalized the manuscript. All authors read and approved the final manuscript.
